# From air to insight: the evolution of airborne DNA sequencing technologies

**DOI:** 10.1099/mic.0.001564

**Published:** 2025-05-28

**Authors:** Mia F. G. Berelson, Darren Heavens, Paul Nicholson, Matthew D. Clark, Richard M. Leggett

**Affiliations:** 1 Earlham Institute, Norwich Research Park, Norwich NR4 7UZ, UK; 2 John Innes Centre, Norwich Research Park, Norwich NR4 7UH, UK; 3 Natural History Museum, London SW7 5BD, UK; 4 Centre for Microbial Interactions, Norwich Research Park, Norwich NR4 7UG, UK

**Keywords:** air, eDNA, metabarcoding, metagenomics, microbiome, next-generation sequencing (NGS)

## Abstract

Historically, the analysis of airborne biological organisms relied on microscopy and culture-based techniques. However, technological advances such as PCR and next-generation sequencing now provide researchers with the ability to gather vast amounts of data on airborne environmental DNA (eDNA). Studies typically involve capturing airborne biological material, followed by nucleic acid extraction, library preparation, sequencing and taxonomic identification to characterize the eDNA at a given location. These methods have diverse applications, including pathogen detection in agriculture and human health, air quality monitoring, bioterrorism detection and biodiversity monitoring. A variety of methods are used for airborne eDNA analysis, as no single pipeline meets all needs. This review outlines current methods for sampling, extraction, sequencing and bioinformatic analysis, highlighting how different approaches can influence the resulting data and their suitability for specific use cases. It also explores current applications of airborne eDNA sampling and identifies research gaps in the field.

## Introduction

Airborne microbes exert several important influences over our lives, including the spread of human, animal and plant diseases [1,4] and even contributing to weather events as airborne microbes influence cloud formation by acting as ice-nucleating particles [5]. Airborne eDNA can come from multiple sources, including bacteria, fungi and viruses in the air, alongside additional taxa such as vertebrates (as they shed skin etc.) [6] and plants releasing pollen or spores [7].

Airborne eDNA exhibits temporal variation on both short and long timescales, ranging from hourly fluctuations [8] to seasonal changes [9,10]. Given the dynamic nature of airflow and the influence of land use on airborne eDNA [11], it is likely that eDNA varies not only over hours and days but also over seconds, minutes, decades and centuries. Spatial variation also occurs in airborne eDNA, both vertically within the stratosphere [12,13] and horizontally across cities and continents [10,14], with species identified often indicative of land use [15].

Earlier airborne microbiota studies relied on passive spore traps, and pollen and fungi were identified through culturing and microscopy. However, these methods were labour-intensive and unable to capture the full diversity of organisms present. Recent advances have led to the development of devices capable of sampling air at rates of thousands of litres per minute (e.g. SASS 4100), greatly increasing the amount of airborne particles captured. With improved samplers, DNA extraction and amplification methods, low-input sequencing library preparation and the high sensitivity of next-generation sequencing (NGS), it is now possible to sequence amplified DNA from just 60 s of collection [16]. This increased sensitivity enables faster methods and the identification of more species [17,18]. As a result, publications on airborne eDNA have increased, with examples including tracking the spread of SARS-CoV-2 in hospitals [19], monitoring endangered species [6,20], preventing forest disease outbreaks [21] and real-time pollen monitoring [8,22].

**Fig. 1.**
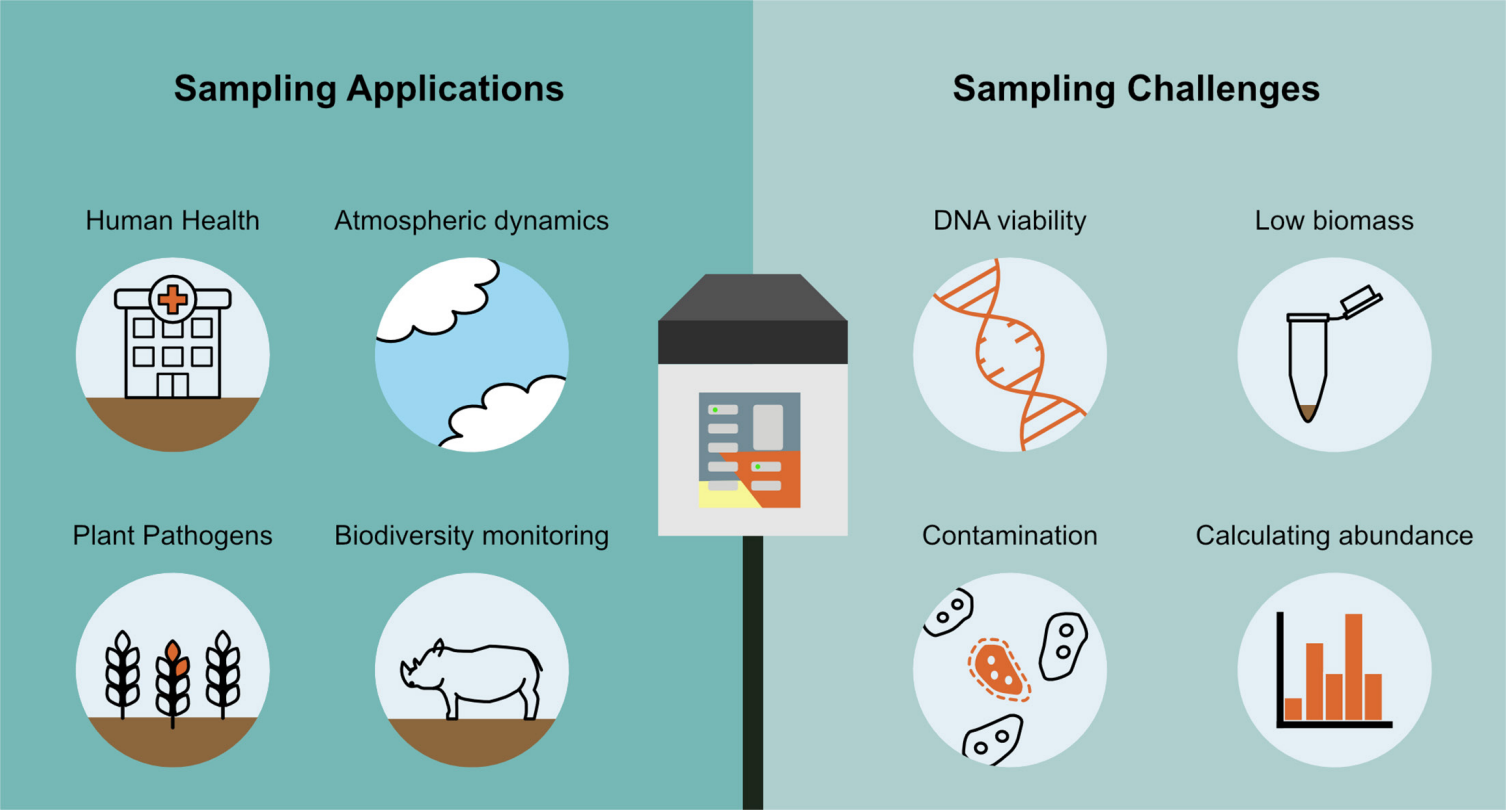
Pictorial representation of applications and challenges of air sampling for eDNA analysis.

This review outlines the end-to-end process of airborne eDNA analysis, describing each stage from sampling to DNA extraction, sequencing and bioinformatic analysis. Furthermore, knowledge gaps, current applications and potential developments within the field are discussed (Fig. 1).

## Overview of airborne eDNA analysis

**Fig. 2.**
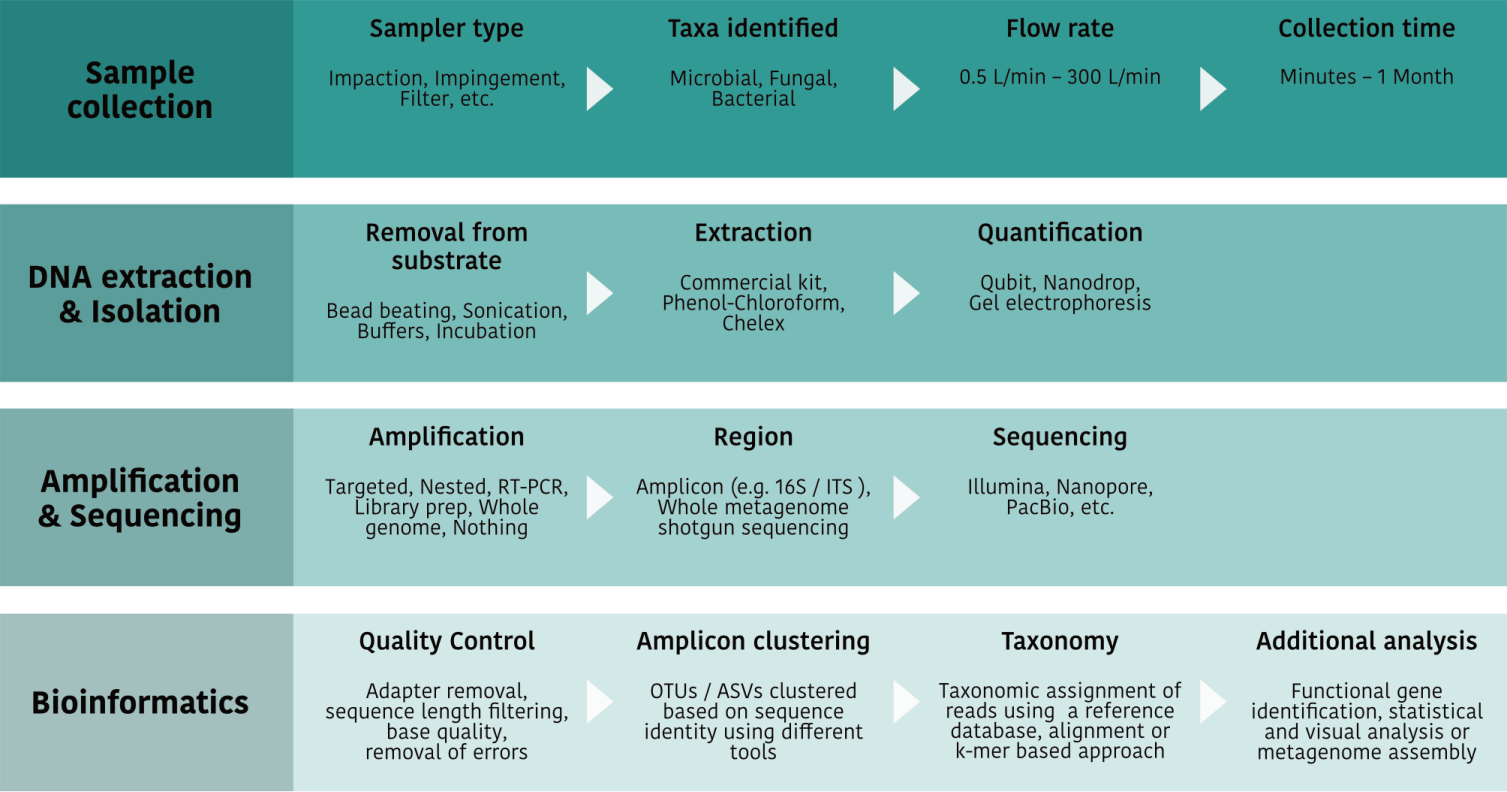
Airborne eDNA analysis pipelines typically comprise four stages: sample collection, DNA extraction and isolation, amplification and sequencing and bioinformatics. Each stage includes several steps with different options depending on the study’s research objectives.

Airborne eDNA analysis typically involves four stages: sample collection, DNA extraction and isolation, amplification, sequencing and bioinformatic processing. The studies reviewed in this article follow this general pipeline (Fig. 2), though the specific method used at each stage can vary depending on factors such as study goals, available resources and equipment. For example, a study conducted in an area with low biomass may require longer sample collection (e.g. a week) to obtain sufficient DNA, whereas a study focused on tracking temporal changes to airborne eDNA in an urban setting might collect several short samples (ranging from 1 min to 1 h) over a single day.

Due to the complex nature of eDNA processing and analysis, the methods employed at each stage will introduce biases in the detection, identification and quantification of taxa. It is crucial to consider these biases when comparing airborne eDNA studies and to account for them before concluding.

### Air collection

Air collection is the first critical step, as enough DNA must be obtained for sequencing and it needs to be sufficient to accurately represent the true composition of the air microbiome. Factors such as the sampler used, collection duration, flow rate and sampler height can all influence the detected airborne eDNA. These variables are interconnected, as samplers have limited collection times and flow rates, and their efficiency can vary.

#### Type of sampler

Air samplers primarily operate through impaction, impingement or filtering [23]. They can be either passive, with no air flow, or active, where air is drawn into the sampler. The advantages and disadvantages of these mechanisms are outlined in Table 1. Samplers differ in efficiency, flow rate and collection surfaces, all of which can influence the type and diversity of organisms detected. Consequently, the choice of a sampler should be tailored to the specific research question. Additionally, the sampler attributes can affect downstream DNA extraction and sequencing; active samplers with high flow rates will collect more DNA.

**Table 1.** Common commercially available air samplers, after [23,24]

Category	Sampler available	Description	Advantage	Disadvantage
**Impaction**	Burkard 7 Day sampler,Andersen sampler,MicroPEM,AirPort MD8 364 device	Particles are impacted onto a surface (adhesive, agar, membrane filter etc.) transverse to airflow	Can select which particle size is recovered by adjusting the flow rateDirect collection onto agar for viability testing	Particle bounce can reduce collection efficiency
**Impingement/cyclone**	Bertin Coriolis, SKC Biosampler, VIVAS	Particles become suspended in a cyclone of air/liquid	Higher flow rates than filter samplers	Lower collection efficiency compared to filter samplers
**Filtering**	Thermo Scientific MFC-PM10 High Volume Air Sampler, Innovaprep Bobcat/Cub, SASS 3100/4100 Dry Air Sampler	Air is passed through a filter (fibrous, membrane or flat), which may include electrostatically charged filters (‘electret’)	Easy-to-use filter and recovery systemElectret filters can collect particles smaller than the pore size	The choice of filter size determines collection efficiency for various particle sizesNot all particles captured (could also be an advantage)

Since this review focusses on providing an overview of approaches that combine DNA sequencing with air sampling to identify airborne taxa, we do not extensively discuss the merits of different sampler models. Instead, we refer the reader to existing studies that compare air sampler attributes [23,24] and those focused on the specific sampling mechanism, such as passive samplers [25] and rotating arm impaction [26]. Below, we offer some general observations on the attributes of the three types of samplers.

#### Air sampler comparison

The ideal air sampler would capture 100% of the particles in the air that pass through its collector, representing the full diversity of airborne eDNA with enough sensitivity to detect even the rarest taxa. It would also allow tuning of the captured particle sizes according to the specific requirements of the experiment. However, such a device does not yet exist. Therefore, it is crucial to evaluate commercially available samplers based on their particle collection efficiency, measured eDNA diversity and sensitivity. Collection efficiency can be discerned by assessing the DNA concentration of a sample or, in the case of bacteria, by evaluating the 16S rRNA gene copy number. The ability of a sampler to fully capture organism diversity, including rarer taxa, is determined after DNA sequencing during the analysis phase.

One study comparing the collection efficiency of liquid impingement and filter impaction samplers used 16S rRNA gene copy abundance to assess DNA yield. The results showed that filter impaction recovered over an order of magnitude more gene copies than impingement [24]. This higher DNA yield is likely due to the increased particle retention efficiency of filter samplers. Another factor influencing sampler yield may be the effect of collection surfaces on downstream processes. In a separate experiment, researchers compared the DNA yield from culture and non-culture collections using membrane filtration, liquid impingement and an electrostatic collector (EC). The EC was the most efficient method, likely due to the much higher flow rate, i.e. 10–100 times greater than the other samplers tested [27].

Even when using the same sampler, the choice of capture material can affect collection efficiency. A comparison of five different membrane filters revealed significant differences in DNA recovery efficiency, with sequencing results showing variations in the diversity of recovered taxa [28]. Another study compared microbial communities detected by five air samplers and settled dust, finding that sampler type had a greater influence on microbial composition than collection location [29]. Differences in flow rates, particle size cut-offs, height and collection media likely contributed to this variation.

Studies on sampler sensitivity are less common in the literature, as they require controlled environments such as clean rooms. A comparison of two impingement samplers found that the VIVAS had higher sensitivity and detected viruses not observed in BioSampler collections [30]. Another study comparing eight samplers found that high flow rate samplers collected the most viruses, but lower flow rate samplers, including the VIVAS, more accurately determined airborne viral concentrations [31].

Research on bacterial pathogen detection also highlights differences between samplers. A study comparing impaction (AirPort MD8), impingement (BioSampler) and cyclone (Coriolis Micro) samplers found that all detected *Coxiella burnetii*, but their effectiveness varied at different concentrations [32]. Similarly, a study investigating airborne vertebrate DNA found that the Burkard spore trap recovered higher numbers of vertebrate species compared to cyclone samplers (Coriolis μ and Burkard multi-vial cyclone) [33].

#### Sampling volume

Collecting sufficient DNA is essential for downstream extraction, sequencing and analysis. However, collection duration and volume depend on experimental considerations, such as capturing all species present or monitoring temporal changes in airborne eDNA. Increasing air volume through a higher flow rate or longer collection time has been shown to increase DNA yield [24,34] and likely increases the number of identified species, though published data on this relationship remain limited.

To comprehensively characterize all taxa present in airborne eDNA, collection periods may need to span days or weeks, particularly in low biomass locations. For example, in a study conducted at polar sites, samples collected for a fortnight contained 16S rRNA gene concentrations comparable to negative controls, suggesting that significantly longer collection times would be necessary for sufficient DNA recovery [35].

Species accumulation curves can help determine whether most taxa in a given area have been captured. The number of identified taxa generally increases with sampling time, reflecting the complex and dynamic nature of airborne eDNA. In one study, samples collected for 1–403 min showed no plateau in species richness when plotted as an accumulation curve [16]. As a result, studies aiming to capture the full diversity of airborne eDNA often employ 7-day collection periods [21,36]. For studies investigating temporal changes in airborne eDNA, shorter ‘snapshot’ collections may be required. Higher flow rate samplers are particularly useful in these cases, as they can collect sufficient DNA within a short period. Notably, a personal impingement sampler has been developed that can detect microbes from just 10-s samples, albeit using quantitative PCR (qPCR) [37].

#### Sampler placement and environmental considerations

Choosing where to place the sampler is a critical decision in aerosol sampling experiments as factors such as height above ground, wind direction and surrounding habitat all influence what is detected. There is no universally optimal placement, but positioning can be guided by knowledge of the local area, meteorological parameters and the biological system of interest [38].

Indoor sampler placement depends on the study’s objectives. For instance, samplers may be placed in bedrooms to monitor overnight allergens, near infected patients to monitor airborne pathogens or in hospital corridors to study the spread of microbes of clinical concern [39,41]. A study comparing indoor samplers at different heights found no significant differences in microbiome composition or diversity, likely because indoor air is contained within the environment and relatively homogenous [40].

Outdoor samplers should also be positioned based on the study’s focus. For example, to detect spores from a soil-borne pathogen, samplers should be placed low to the ground [38]. Conversely, samplers positioned above the floral canopy are more likely to detect airborne pathogens arriving from distant locations [23]. One study comparing rooftop and in-field samples found that fungal species richness was higher in rooftop samples than in those placed above the crop canopy [42]. This is because samplers near the crop canopy primarily capture local spores, whereas those 10–30 m above ground collect a more mixed sample containing biological material from further afield [23].

Topography plays an integral role in airborne eDNA dispersion. Valleys and mountains influence wind speed and direction, affecting how biological particles travel. Large structures create ‘quiet zones’ where air movement is reduced; these zones can reach up to twenty times the height of the structure [38].

#### Time of sample collection

The timing of air collections is also extremely important, as both the time of day and season can significantly affect airborne eDNA composition. Knowing when a sample was collected is essential for accurate interpretation and comparison across airborne eDNA studies. Seasonal shifts in airborne eDNA are well-documented, with specific taxonomic groups more abundant at different times of year. For example, pollen from flowering trees and plants is more prevalent in spring, while grasses and mosses, along with wood-rotting fungi, are more abundant in summer. In autumn, fungal spores dominate, and winter is marked by reduced plant pollen and the presence of cold-tolerant bacteria [9,43, 44].

In addition to seasonal changes, airborne eDNA composition is known to oscillate throughout the day with changes in UV, temperature and humidity. These environmental factors can create consistent daily patterns, such as with spore release. For example, powdery mildew is often abundant at midday, tropical fungi sporulate overnight (or before sunrise) and *Alternaria* spp*.* are more prevalent in the afternoon [45,46]. Other shifts in composition may be more spontaneous. For example, rainfall can affect the composition of airborne bacteria and fungi by washing out existing particles while dispersing new microbial particles into the air through splash effects [47,48]. High wind speeds increase microbiome diversity [49], while wind direction can affect the microbiota present [50] and humidity triggers spore release in many fungi [51].

Diurnal differences have also been observed, and one study within an urban environment found human pathogenic bacteria to be more abundant during the day than at night [52]. Another study in Siberia recorded that the fungal community has the highest relative abundance and diversity at night and decreases during the day, whereas the bacterial community peaked in the evening and decreased at night [43].

These dynamics highlight the importance of recording metadata – such as local weather conditions and the time of sample collection – to enhance data interpretation. Incorporating this metadata into analyses can reveal how different taxa respond to diverse environmental conditions, providing a more comprehensive view of airborne eDNA dynamics.

#### Particle size

Particle size is a key factor in airborne eDNA dispersal, influencing both residence time in the air and sampling efficiency. Airborne biological particles can be in the nanometre range, but typically range from 0.65 to 12 µm in many environments [53], with smaller particles remaining suspended longer and larger ones settling more quickly [54]. To improve capture rates, organisms with larger particles and shorter airborne times may require sampling closer to the source, for extended durations, or during periods when they are more likely to be airborne due to environmental or temporal factors. Additionally, different samplers are optimized for collecting specific particle sizes, making sampler selection critical. While literature on airborne particle size and eDNA monitoring is limited, Mahaffee *et al*. [38] provide a review on optimizing fungal spore detection, and several studies compare airborne community composition across different particle size fractions [48,55,58].

### DNA extraction and isolation

Following air collection, the next steps are to lyse cells and spores etc. from the samples, extract DNA and isolate the DNA from other biological matter – these processes can introduce bias into results. Most studies use a commercially available extraction kit with modifications such as additional incubation or filtering steps. Limited studies are comparing DNA extraction protocols for air samples, and they are often restricted to a specific sampler or collection medium.

Obtaining sufficient high-quality DNA from airborne samples is essential, as it affects sequencing, taxonomic assignment and other downstream analyses. Low DNA yields can result in poor sequencing data, while degraded DNA may lead to reductions in yield, misidentifications or increased errors. Because of this, it is important to choose an extraction method that maximizes DNA yield while preserving quality for accurate results.

Prior to DNA extraction, samples usually require concentrating either through filtration or centrifugation. A published comparison of filtration and centrifugation of collection liquid used the Internal Transcribed Spacer (ITS) gene to compare identified fungal communities. Filtration yielded three orders of magnitude more DNA than centrifugation and identified taxa absent in centrifuged samples [18]. These findings suggest filtration provides a more comprehensive fungal community than centrifugation for liquid samples.

Once concentrated, cells are lysed, and DNA is extracted, typically using commercially available microbiome extraction kits. However, none yet exist that are specifically designed for air samples (unlike, e.g. soil, for which specific kits exist). A comparison of the efficacy of four extraction kits targeted to specific sample types but tested on air samples collected onto quartz filters found that water and soil kits produced higher 16S rRNA gene copy numbers than blood or human tissue kits. Notably, the study did not conduct taxonomic comparisons to measure the sensitivity of the kits or the sample diversity captured [35]. An additional complication arises from contamination by the so-called kitome, which is the genetic material already present in these extraction kits, which may be misconstrued as DNA from the sample [59].

Cells of certain species are easier to lyse, more prone to degradation or less likely to adhere to tube walls, leading to their disproportionately high abundance in the DNA extract [18,60]. For example, researchers found that DNA extracted with the Zymo Research Fungal/Bacteria DNA Microprep Kit from a mock community resulted in over- and under-represented species and that sporulating fungi were generally underrepresented [61]. Inevitably, bias is introduced during DNA extraction, and the observed taxonomic abundance is different from reality. Another study used captured aerosol samples to compare three extraction protocols with different incubation and lysis steps. The authors found that among the top 10 most prevalent species of each sample, only 5 were shared between the extraction methods, showing that the extraction protocol has a large influence on the species identified. This study also used centrifugation to concentrate the DNA and then separately analysed the pellet and supernatant, finding they had substantially different taxonomic composition [62].

These same studies compared the read numbers of different taxa from samples to highlight how bias can affect results, as different protocols may detect species at varying prevalence. While some level of bias is inevitable in metagenomic studies, minimizing it where possible is crucial. Equally important is maintaining consistency in sample handling and DNA extraction protocols throughout a project to ensure any remaining bias remains constant and does not introduce additional variability.

Other more comprehensive extraction protocols have been developed for metagenomic samples, such as the ‘three peaks’ faecal DNA extraction method, which uses three different lysis steps (chemical, enzymatic and mechanical) targeted towards different organisms within the sample while retaining long DNA molecules [63]. Although not routinely used on air samples, it has the potential to further optimize extraction by finding a better balance between lysis and DNA degradation.

### Amplification and sequencing

After extraction, it is common for researchers to deploy metabarcoding, which involves PCR primer-based amplification of a barcoding gene or, less frequently, whole-genome shotgun sequencing (WGS), with or without whole-genome amplification. Whether to use metabarcoding or WGS depends on factors such as the required taxonomic resolution, time, cost and the specific research question. For example, a study investigating bacterial community composition may use 16S amplicon sequencing, while another looking at antimicrobial resistance (AMR) gene abundance would opt for WGS to capture a broad range of genes present in airborne eDNA.

#### PCR for known species

PCR approaches can be employed to target specific species, and this is particularly valuable in scenarios where only a few species are of interest and precise identification is crucial. For example, PCR has been used in the detection of *Campylobacter* spp. from air filters in poultry houses [2] and for the detection of multiple airborne microbes simultaneously from samples collected with a prototype personal bioaerosol sampler [37]. These methods are only able to detect specific species, but WGS or metabarcoding would be necessary to identify all potential taxa present (using reference genomes). Despite these limitations, PCR provides a rapid and specific method for detecting pathogens in air samples, thereby complementing broader metagenomic approaches, such as [2], where WGS was conducted alongside PCR.

#### Metabarcoding

Metabarcoding uses primers to target highly conserved genomic regions that contain short variable sequences, which can be used to distinguish taxa – sometimes to the level of species, but often to higher taxonomic levels or to operational taxonomic units (OTUs) representative of multiple species. For bacterial and fungal communities, this is often the 16S and ITS rDNA region, and amplicon approaches based on these regions have been successfully used to monitor airborne eDNA [11,21, 24, 40]. Metabarcoding is often chosen as it is cheaper than WGS, and the use of PCR means it is more robust to low amounts of DNA [64]. However, PCR can impact the detected microbial diversity by inducing bias during amplification, while taxonomic identification from amplicons has lower resolution than with WGS data [34,65]. Banchi *et al*. [61] have reviewed the use of metabarcoding in aerobiological studies in detail, so here we will discuss how amplification can affect the detected taxa. A comparison of the ITS1 and ITS2 regions for metabarcoding of airborne fungal spores found the fungal community composition to be different depending on the region amplified [66]. In addition, some species are only identifiable from one of the regions, for example, in fungi, *Armillaria* spp. only with the ITS1 marker and *Hymenoscyphus fraxineus* and *Melampsora larici-populina* only with the ITS2 marker [34]. Another study compared five different ITS primers with the same sample and detected different fungal communities with each set, with each primer pair able to detect ~50% of the fungal diversity which increased to 70–80%, when results from two different primer sets were combined [67]. In bacteria, a comparison of 16S and 12S amplicon sequencing identified different unique species [6]. Thus, airborne eDNA studies that use one metabarcoding region do not identify some unique species, limiting the characterization of airborne eDNA.

Further limitations of metabarcoding come from limited taxonomic resolution and bias in the detected community composition. Amplicon reads are often classified to higher taxonomic levels than species, and this has been demonstrated to be the case with fungi, bacteria and vertebrates [6,40, 68].

As sequencing technologies advance, long-read metabarcoding is gaining traction, offering full-length marker gene sequences rather than shorter regions, thereby improving taxonomic resolution [69,70]. The decreasing error rate of nanopore sequencing and the reducing cost of PacBio sequencing make these methods increasingly viable [71]. However, this data needs to be processed differently from other metabarcoding data [72]. There is currently a lack of published studies applying long-read metabarcoding to airborne eDNA, but it holds great potential for the future.

#### Bias introduced during amplicon sequencing

Biases arising from amplification within metagenomic studies are well documented in the literature [60,73], with taxa being over- and under-represented in mixed samples after 16S PCR [60]. Several factors contribute to this bias, including primer choice, annealing temperature and the number of amplification cycles [73,77]. Increasing the number of PCR cycles has been shown to reduce community richness by ~4–10-fold, as taxa with more efficient primer binding sites are preferentially amplified, leading to an overrepresentation of some groups and the loss of others [75,77].

Beyond amplification bias, copy number variation can also skew relative abundance estimates in amplicon sequencing because within a metagenomic sample, organisms have different numbers of the amplified gene. For example, in bacteria, the 16S gene copy number varies from 1 to 15 between species [78]. Organisms with a higher copy number will, therefore, have an inflated abundance in the sample.

Additionally, even single-base mismatches between primers and template DNA can skew amplification efficiency. These mismatches have been shown to cause preferential amplification of certain taxa, altering the relative abundances in the detected community. They can also affect taxonomic resolution by changing amplicon length, which impacts classification accuracy [74].

Although most metagenomic studies addressing these issues have focused on microbial mock communities or gut and stool samples, these findings are likely transferable to airborne eDNA studies. Given these challenges, it is unlikely that a single set of PCR conditions can amplify all organisms equally efficiently. For a more in-depth discussion of PCR biases in metabarcoding, readers are referred to recent reviews on this topic [79,80].

#### Whole-genome sequencing

WGS can detect all kingdoms simultaneously and be used for species and functional gene identification. In the absence of other constraints, it may be considered a preferable method for profiling airborne eDNA as it identifies a broader range of species than metabarcoding [4,39, 43, 62, 81,83].

Another advantage of WGS is that it typically requires little or no amplification, reducing biases associated with PCR. As a result, relative abundance estimates from WGS data are typically closer to the true community composition than with amplicon sequencing. However, a drawback of using WGS over amplicon approaches is that it requires a larger quantity of starting DNA, which can be a limitation when working with air samples, which typically have low biomass. One approach to mitigate this challenge is to reduce reaction volumes during library preparation, as demonstrated in a study where a nanopore sequencing run was successfully conducted using just 6.25% of the recommended DNA input [84].

A study comparing 16S metabarcoding and WGS of air samples found both methods to converge at the phylum level but did not produce significant agreement at the species level, likely due to the 16S providing a less accurate result at the species level [34]. Additionally, WGS enables the simultaneous comparison of bacterial and fungal species, as both groups can be sequenced together, ensuring that their relative abundances are directly comparable. In contrast, using different primers for amplification in metabarcoding results in disparities that make abundance comparisons between fungi and bacteria more unreliable. A further benefit of WGS is the ability to assess the functional diversity of airborne eDNA – for example, to see the presence of detoxification, antibiotic resistance and virulence genes. However, a weakness of WGS is the less comprehensive reference databases that are currently available for classifying taxa – for example, the latest UNITE release (v10) contains ITS sequences representing ~11 times more fungal species than the number of fungal species with reference genomes available in the National Center for Biotechnology Information (NCBI) RefSeq (release 228).

### Bioinformatics

**Fig. 3.**
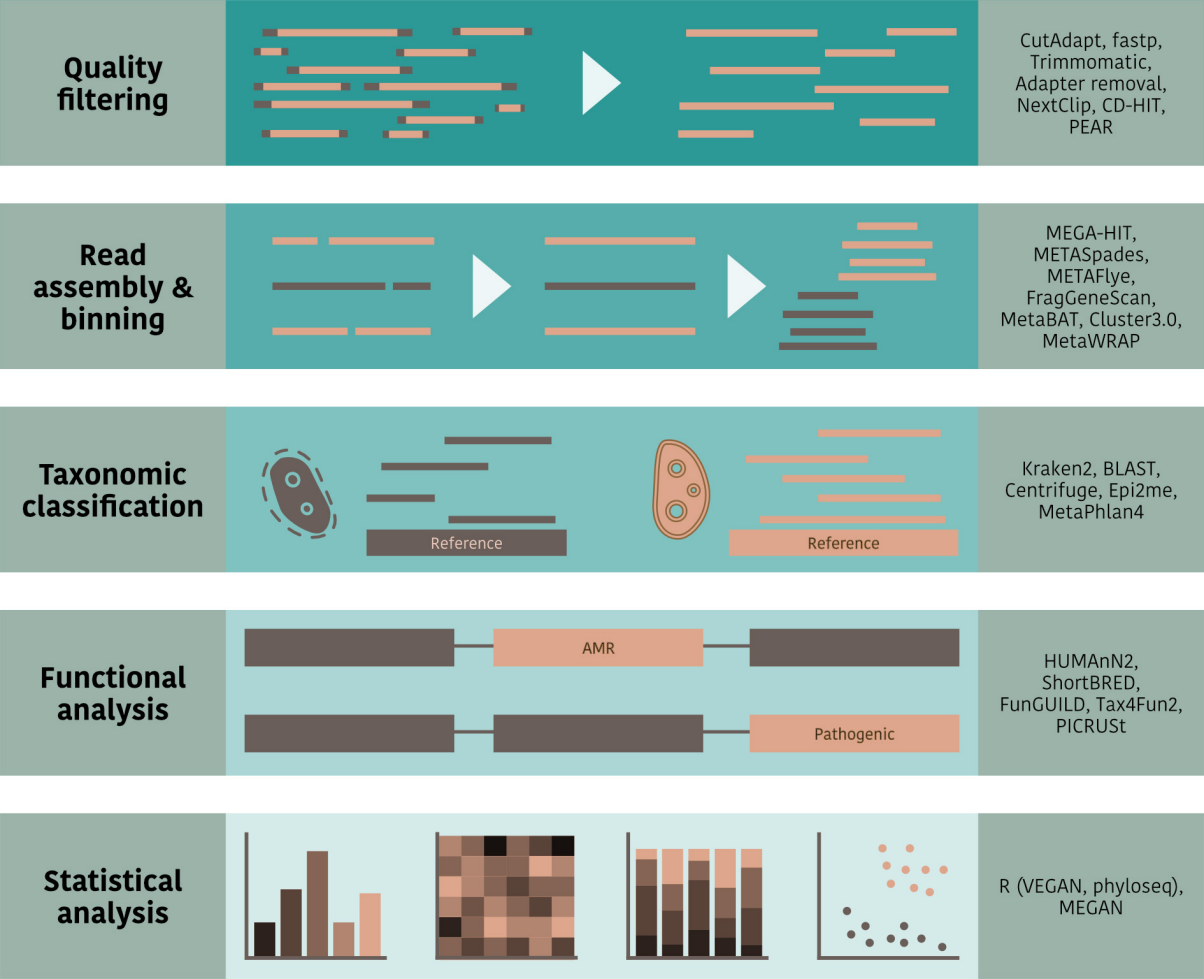
Stages in bioinformatic analysis for sequence data generated from air samples. On the right of each row is listed a selection of commonly used tools. Further details of the tools can be found in Table 2.

In common with other metagenomic studies, there are typically two key questions that are asked of the data in an air eDNA study: ‘What is there?’ (taxonomic classification) and ‘What is it doing?’ (functional analysis). Here, we briefly outline the bioinformatic steps involved and how they have been applied to air samples. Fig. 3 summarizes these steps along with commonly used tools for each stage, references for which can be found in Table 2. For more detailed discussions on eDNA data processing and its impact on taxonomic and functional classification in amplicon and WGS studies, see [85,89].

**Table 2.** Commonly used tools for bioinformatic processing. A more in-depth discussion can be found in [153] and in some of the review papers cited in the first column

Stage	Specific purpose	Tool name	Reference
**Quality filtering**See also [90]	Remove adaptor sequences	CutAdapt	[154]
		fastp	[155]
		Trimmomatic	[156]
		Adapter removal	[157]
	Remove duplicated reads	NextClip	[158]
		CD-HIT	[159]
	Map reads to human reference and remove from sequences	Minimap2	[160]
		BowTie	[161]
	Merge paired-end reads	PEAR	[162]
**Read assembly**See also review of assembly tools [93,163]	Generate longer contigs from short reads to generate MAGs	MEGAHIT	[164]
		metaSPAdes	[165]
		metaFlye	[166]
		metaMDGB	[167]
	Identify fragmented genes in short reads	FragGeneScan	[168]
**Binning**See also the discussion of binning tools for WGS [94] and amplicon [96]	Group contigs together based on sequence similarity	MetaBAT	[169]
		MaxBin	[170]
		CONCOCT	[171]
		Cluster3.0/Pycluster	[172]
		MetaWRAP	[173]
**Taxonomic classification**See also review of classification tools [174]	Identify taxa from sequences by alignment to reference genomes	minimap2	[160]
		BLAST	[97]
		BWA	[175]
	Identify taxa from sequences with a *k*-mer-based approach	Kraken2	[98]
		Centrifuge	[176]
		MASH	[177]
	Identify taxa from sequences with a marker-gene approach	MetaPhlan4	[99]
**Functional analysis**	Identify genes with known functions	HUMAnN2	[178]
		ShortBRED	[179]
		FunGuild	[115]
		Tax4Fun2	[180]
		PICRUSt	[181]
**Statistical analysis**See also [121,123]	Perform abundance and diversity measures	VEGAN in R	[182]
		Phyloseq in R	[183]
**Visualization**	Plot graphs of the analysis	Ggplot2 in R	[184]
**Multiple stages**	Used in combination with blast to classify taxa and can be used to carry out statistical analysis and visualization	MEGAN and MALT	[185,186]
	Bioinformatic workflows specific to Oxford Nanopore reads (basecalling, alignment and assembly)	Epi2me	https://labs.epi2me.io/
	Real-time classification and AMR analysis for nanopore sequencing	MARTi	[187]
	Amplicon-specific software tool that requires fastq files as inputs, which are then quality filtered, clustered into ASVs and assigned to taxa	DADA2	[188]
	A microbiome platform that is open-source, free and community-developed; the tool records the steps in bioinformatic pipelines to ensure they are reproducible and reusable by others	Quantitative Insights Into Microbial Ecology (QIIME 2)	https://qiime2.org/
	A tool designed to bin >100 bp reads; can also assign taxonomy, build phylogenetic trees, perform functional analysis and compare datasets	MetaBin web server	[189]

#### Quality filtering

Quality filtering is a necessary pre-processing step and will help to prevent misidentification from alignment of low-quality reads with greater numbers of errors. Initially, sequencing adaptors are removed, and paired-end reads are merged where applicable and libraries with multiple samples are demultiplexed. Reads are then filtered on a variety of metrics but most often on length and quality score, the specific thresholds depending on the sequencing platform and study objectives. A review of the importance of quality filtering sequence data for downstream analysis is available at [90].

#### WGS read assembly

An additional optional step before taxonomic classification of WGS reads is assembly, in which shorter reads are combined into longer contigs to improve taxonomic precision by providing more genomic context, particularly for closely related species (see Ayling *et al*. [91] for a review). This approach is widely used in metagenomics and has been applied to airborne eDNA studies, enabling gene and protein prediction, and alignment to reference databases for fungal spore identification and species confirmation [4,83, 92]. Following assembly, contigs may be grouped or ‘binned’ into genome-level metagenome-assembled genomes (MAGs) for further analysis. A large number of tools are available to facilitate this process, and in-depth discussions can be found in [93,95].

#### Amplicon binning

Metabarcoded samples are initially clustered into OTUs or amplicon sequence variants (ASVs) prior to taxonomic identification. OTUs are defined based on a sequence similarity threshold, which helps reduce the impact of sequencing errors but may also group closely related species together. In contrast, ASVs distinguish true biological variation from sequencing errors by incorporating sequence abundance, allowing for higher resolution at the cost of potentially discarding low-abundance sequences as noise.

While ASVs generally offer greater resolution, especially in terms of species-level identification, they can also be more susceptible to noise when dealing with rare taxa, which is particularly relevant in airborne eDNA, where low-abundance organisms may be underrepresented. For example, in airborne bacterial pathogen studies, assembly methods have successfully enhanced 16S read clustering into OTUs, improving species-level classification [52].

The choice of clustering approach (OTU/ASV) and parameters used will affect the community composition downstream analysis and is, therefore, an important consideration in study design. For example, a comparison of OTUs and ASVs clustering on the same dataset found little agreement at the species level but more consistency at the genus level [21], highlighting how the clustering approach can affect the result.

For a more detailed discussion of denoising and clustering methods, Hakimzadeh *et al*. [96] provide an in-depth analysis of these methodologies and their application to metabarcoding data.

#### Taxonomic classification

Clustered OTUs/ASVs or WGS reads are then mapped to reference databases for taxonomic identification, and the methods used for this vary. Historically, taxonomic identification was carried out with alignment-based approaches using blast [97] or other blast-like tools. However, as reference databases have expanded, alignment methods have become increasingly compute and time hungry, leading to many adopting more computationally efficient tools using *k*-mer-based strategies such as Kraken2 [98], or marker gene approaches like MetaPhlan4 [99]. These *k*-mer-based approaches typically require an up-to-date taxonomy of the reference database to be provided, and assignments may be inaccurate if this taxonomy is incorrect.

The choice of database is crucial for any alignment or *k*-mer-based analysis, as these approaches can only detect species that are represented in the database. If an organism is missing, reads may be misassigned to a closely related species, leading to false positives. Studies have shown that the detected community composition can vary greatly depending on the database used [100]. Many databases lack sequences for a substantial portion of microbiome diversity, particularly in airborne eDNA studies, where many microbial taxa are unculturable. This leads to species being classified at higher taxonomic levels or misassigned to closely related taxa. Errors in reference data, such as incorrect annotations, poor quality references or contaminated genomes, can further skew results.

For classification approaches utilizing lowest common ancestor (LCA) assignment (in which reads are placed at the lowest point in the taxonomy consistent with all ‘good’ matches), the increasing size of databases has been shown to increase the number of classified reads but make species-level taxonomic assignment more difficult. This occurs because the number of new species being uploaded to databases far outpaces the addition of new genera, increasing the likelihood of ambiguity at finer taxonomic levels. As a result, the LCA approach is more likely than it was to assign reads at the genus level rather than the species level [101]. Alongside this, the increasing size of databases makes greater demands on computing resources and makes it difficult to keep them up to date. For a more in-depth discussion of these reference databases’ problems, see [102].

Airborne eDNA studies often map WGS reads to large databases like NCBI’s nucleotide or non-redundant databases [6,33, 39, 43, 44, 103], but some opt for smaller, curated databases tailored to specific taxonomic groups and research questions. Metabarcoding studies use gene-specific databases (e.g. ITS, 16S and 18S) for quicker analysis and fewer misalignments. For instance, the UNITE database [104], which only contains ITS sequences, has been used to study airborne *Alternaria* spp. [105], rice pathogens [50] and fungal communities in forests [12]. In bacterial metabarcoding, the silva rRNA database, which includes 16S and 18S sequences [106], has been used to identify urban airborne bacteria in Tokyo [49], analyse live airborne micro-organisms [107] and identify airborne bacteria over the ocean [108].

Some study authors create custom databases, such as using viral reference genomes from NCBI to investigate seasonal changes in the airborne virome [109]. Similarly, the PHI-base (plant–host interaction) database [110] was used to generate a custom pathogen database in order to identify airborne plant pathogens [4].

Taxonomic assignments, particularly to species or strain level, should always be treated with a degree of caution. Post-filtering steps can be implemented to increase certainty. For example, a study of airborne eDNA in West Siberia reduced incorrect mappings by only accepting species-level classification when enough reads aligned uniquely to a single species [43]. This threshold will differ between studies, depending on factors like amplification and sequencing depth. Studies that do not amplify DNA may identify rare species with fewer reads than the above study, and disregarding these reads could result in the loss of measured diversity.

#### Functional analysis

Alongside taxonomic classification, functional analysis of airborne eDNA can provide insights into the activity of the organisms present. Metabarcoding genes can be used only to *infer* the function of present taxa, while WGS can be used to identify specific genes or transcripts. This means that WGS offers greater functional insights, assuming sequencing depth and DNA yield are high enough – though these both can be limiting factors in airborne eDNA studies

Functional analysis in air studies has largely focussed on the presence of AMR genes, using databases such as Comprehensive Antibiotic Resistance Database [111] or Structured Antibiotic Resistance Gene [112]. For example, studies have been conducted on the presence of airborne AMR genes using WGS on public transport [81] and hospitals [113] and real-time qPCR on samples collected from poultry sheds [114].

Functional analysis has also identified plant pathogenic potential in airborne fungi [21,66] using the ITS region and tools like FunGuild [115]. Similarly, studies on airborne bacteria along the Antarctic coast used 16S rRNA gene analysis to reveal functional profiles related to biosynthesis and metabolism [116]. Additionally, a meta-analysis of global airborne microbiome diversity showed that anthropogenic activities correlate with increased abundance of pathogenicity genes in the air, using 16S data [117]. This aligns with a similar study in China, which found the relative abundance of pathogenic bacteria to increase as air quality decreased, using qPCR of specific genes [118].

There is also the potential to identify other functional genes such as virulence or toxicity within a metagenomic sample, as seen in marine and soil samples [119,120]. But to our knowledge, there exist no published studies applying these approaches to air samples. Examining the functions of airborne microbes can reveal important insights into antimicrobial resistance, pathogenicity and metabolic potential, increasing knowledge on how these communities interact with their environments.

#### Statistical analysis

Once classified, data can be imported into analysis software like R or processed through custom bioinformatic pipelines to assess diversity, uncover patterns or dependencies or discover ecological insights relevant to environmental health and microbial dynamics. Read counts per taxa can be used to calculate alpha and beta diversity for a sample set. There are several reviews discussing the statistical analysis undertaken for microbiome data, which can also be applied to airborne eDNA studies [121,123].

Airborne eDNA studies typically involve other bespoke analyses, for example, detecting specific pathogens [50,124, 125], correlation analysis with weather and environmental data [4,49, 92] or determining how airborne eDNA composition changes with distance from a source such as a waste treatment plant [11] or from land [108].

## Current applications of airborne sampling

Sequencing of airborne eDNA has been applied to a vast range of fields encompassing plant and pathogen detection, high-altitude and oceanic air sampling, human health monitoring and biodiversity estimation (Fig. 1).

### Detection of plants and their pathogens

Airborne eDNA analysis can be used to monitor plant pathogen outbreaks, including within agricultural or forestry systems. Historically, these settings have relied on passive collection setups in which airborne spores land on a sticky tape for analysis with microscopy. This is a slow and labourious process which requires identification expertise, but NGS promises a rapid, affordable and accurate airborne detection methodology.

A comparison of microscopy and metagenomics on the same spore trap samples showed that both methods were able to detect major cereal pathogen genera. However, the metagenomic approach detected pathogenic species that microscopy missed [92]. This demonstrates that DNA sequencing can enhance the knowledge gained from air sampling.

Molecular airborne eDNA techniques also enable large-scale monitoring without the need for labour-intensive surveys. For example, qPCR of known forest pathogens across 12 sites allowed for the detection of wind-dispersed diseases without the extensive effort of typical field surveys [21]. Similarly, a UK study used air sampling and WGS sequencing to identify several agriculturally significant crop pathogens [4]. In another example, airborne rice pathogens, disease severity and environmental conditions were monitored throughout a growing season using both qPCR and ITS metabarcoding [50].

The vast amount of data generated from airborne eDNA studies can be analysed in combination with climate and meteorological data to better understand airborne particulate movement. This approach enhances models of pathogen outbreaks [126] and helps explain how airborne biological particles interact with the atmosphere, leading to ice nucleation [127]. In some cases, the relationship between airborne eDNA and meteorological data can be analysed in reverse. For example, a global network of samplers revealed that annual mean temperature significantly affects spore community composition [10]. Samplers can also collect pollen from the air, as demonstrated in a study involving collection along a distance gradient from a GM maize field in which real-time qPCR was used to determine the ratio of GM to WT pollen and to monitor the dispersal of GM pollen [128].

### High altitude and oceanic air sampling

Sampling airborne eDNA at high altitudes and over oceans presents new opportunities to investigate airborne microbial diversity and expand our knowledge of these environments beyond conventional studies.

The height above ground at which sampling occurs is known to affect the composition of airborne eDNA [129]. Most sampling is conducted near ground level, but some researchers have attempted to classify aerosol composition at much higher altitudes, up to 12,200 m above sea level. These collections require aircraft with air sampling devices that have been adapted for motion at high altitudes and are programmed to collect for specific times at different heights [58,129,131].

Additionally, sampling at high altitudes indicates airborne eDNA composition differs with height above ground; one study found that at heights>1,000 m micro-organisms no longer had a diurnal cycle [129]. In-flight sampling has also been conducted above high-intensity forest fires and has shown that fires can increase microbial concentration and diversity of viable cells [132]. Variation in microbial composition with height needs to be considered when comparing different samples and experiments.

In addition to surveys in the sky, there have been studies of airborne eDNA over the ocean [15,108]. For example, a study of the airborne bacterial community along the Antarctic coast found bacteria which contain genes likely to contribute to ocean-surface growth and survival [116].

Collectively, these findings underscore the necessity of broadening the scope of airborne eDNA research to encompass untapped environments that may exhibit different dynamics than those discussed. The insights gained from such investigations could enhance our understanding of the role micro-organisms play in ecological processes and atmospheric dynamics.

### Sampling for human health

Air sampling has been used in hospitals for various purposes, including establishing microbiome community baselines. These baselines can help identify unusual increases in pathogen abundance in the future [39]. For example, with the emergence of SARS-CoV-2, air samples have been collected from around different parts of hospitals in order to pinpoint high-risk infection zones [1,19, 41].

Wearable air samplers have been used to detect monkeypox in hospitals [133]. In this instance, healthcare workers were wearing PPE while in infectious rooms, but sampling devices could be used in the future to monitor workers’ exposure to infectious diseases. Additionally, air samples taken from rooms of norovirus patients have helped researchers better understand how the virus is transmitted [55]. The same study also found that viable virus particles can survive in the air and linked their presence to how recently a patient had vomited.

While these examples mostly involved amplicon sequencing, whole-genome sequencing of air samples in hospitals and nearby areas has been used to identify the widespread presence of airborne antimicrobial resistance genes and to show that they are more abundant and diverse within hospitals than in nearby locations [113].

These applications illustrate the potential value of air sampling in healthcare settings, by monitoring pathogens, worker exposure and antimicrobial resistance.

### Air sampling for biodiversity estimation

In recent years, molecular methods have become an important tool in the assessment of biodiversity. Airborne eDNA analysis offers a multitude of potential benefits: it will require less labour to monitor large areas, air sampling could occur in inaccessible areas using drones and species that are unlikely to be seen with conventional sampling may still be detected through their DNA. Several proof-of-concept studies have been published.

Vertebrate detection through air sampling has been demonstrated in a zoo with non-native species successfully detected up to hundreds of metres away and with such high sensitivity that the organisms present even in animal feed could be detected [6,20]. Another study compared air samples collected with Burkard spore traps to images from camera traps and found that all species observed with the camera traps were identified using airborne DNA, as well as unique species only seen through air samples [33]. Air sampling in a botanical garden was able to identify 67 plant species out of the 1,585 known to be growing in the garden. The lack of identification of the other species could be due to the plants not releasing pollen at this time, but the authors also suggest that an increase in sampling effort would lead to a higher number of species being detected [7].

Beyond samples collected specifically for biodiversity assessment, filters intended for air quality monitoring have been successfully utilized to extract DNA. In one notable example, researchers measured biodiversity over a 34-year period (1974–2008) using weekly filters from a radionuclide monitoring station that each processed over 100,000 l of air. Deep shotgun sequencing detected over 2,700 genera and correlated land use changes with declines in biodiversity [44]. A similar study also used eDNA extracted from filters in air quality monitoring stations and identified over 180 taxa including vertebrates using amplicon sequencing [36]. These findings indicate that it is possible to assess historical biodiversity when filters are stored under appropriate conditions leveraging pre-existing air monitoring networks.

Collectively, these studies highlight the versatility of air sampling as a tool for biodiversity monitoring, enabling both real-time species detection and retrospective biodiversity assessments.

## Current research gaps

### Is captured DNA viable?

Detecting an organism’s DNA in the air does not necessarily indicate that the organism is still viable, which is an important factor in many airborne pathogen studies. In agricultural settings, costly fungicide applications may only be necessary if the detected spores are viable. Similarly, when studying human pathogens, public health recommendations could depend on whether airborne pathogens pose a real risk to people’s health.

The current best method to assess spore viability is to collect directly onto a growth medium and count the colonies after incubation; the medium can be selective for a specific pathogen [134] or combined with sequencing to identify maximum taxonomic diversity and viability in tandem [58]. However, since culturable fungi make up less than 1% of the total recovered fungal species from airborne eDNA studies [18], the majority of fungal spore viability cannot be assessed in this way.

An alternative method to determine eDNA viability is to use high-throughput sequencing and leverage the fact that active cells typically have higher concentrations of ribosomal RNA. Therefore, species with a high rRNA-to-DNA ratio are more likely to be active than those with a low abundance of rRNA. A study applying this principle to airborne eDNA identified a significant difference in the taxonomic abundance of DNA and RNA communities from the same sample, suggesting that not all the identified species were active [107]. However, this approach may be less applicable to organisms that disperse in a dormant state, such as spores, which remain viable but exhibit little or no rRNA activity.

Researchers are also exploring the possibility of interrogating raw nanopore data to determine if microbes are alive or dead based on the signal produced during sequencing [135]. Since nanopore technology sequences native DNA, it can detect DNA damage and modifications as DNA passes through the pore, represented as signals. Machine learning models interpret these signals, with a recent study training models on DNA from dead and live cells to identify how the signals differ in viable cells [135]. While still in the early stages, this approach could offer another method to assess the proportion of viable organisms in metagenomic samples.

### Low biomass in the air

There is very little biomass present in the air and, therefore, low concentrations of DNA and, especially, RNA, to collect and sequence. A study of the SARS-CoV-2 virus found the concentration of its genome was just 0.87 copies per litre of air, which was judged insufficient for NGS methods but still suitable for Sanger sequencing. Another study collected bacteria and virus-like particles by size and found they were present at ~10^5^ and 10^6^ particles on average per cubic metre of outdoor air [136]. Airborne DNA concentrations also vary seasonally. A study, in Siberia, found a 170-fold increase in DNA yield in the summer compared to winter [43], though such large variations may not be typical in less extreme climates. Another study identified many more sequenced reads in spring compared to autumn, likely due to plant species growing, flowering and releasing pollen in the spring [137].

The concentration of DNA also varies by location: in areas such as Antarctica, there is very little DNA present in the air due to the presence of few living organisms. To overcome this limitation in one Antarctic study, samplers were operated for 2 weeks to collect 16,000 m^3^ of air. The DNA yield, measured by 16S concentration, was still found to be the same as negative controls [35].

Due to the low concentration of airborne DNA, some studies have refined pipelines to increase the concentration of DNA collected, allowing for shorter collection times. For example, one study demonstrated that improvements to the sampling, storage, extraction and nucleic acid analysis steps increased DNA accumulation by 8–170-fold over other airborne eDNA studies [34]. Furthermore, a new DNA-isolation method from the MetaSUB project increased the DNA yield and reported diversity of airborne eDNA from subway samples, but not simple mocks [62]. The MetaSUB method uses a chemical lysis buffer and centrifugation process to remove DNA from the sampler’s filter, followed by enzymatic and mechanical lysis to increase DNA yield.

### Preventing contamination

Airborne eDNA sequencing is inherently at risk from contamination, as the low biomass means that any taxa introduced, even at low quantities, will be retained through standard analysis. Additionally, contamination could be added during the amplification processes necessary for metabarcoding [6]. Contamination is a known concern in eDNA studies, and as such, there are reviews on how to minimize both cross-contamination (between samples) and external contamination (from the laboratory, reagents or researchers) [138,139].

External contamination can be minimized with sterile procedures and managed through the collection of negative controls from all steps of sampling and processing [140]. Negative controls are important as DNA extraction kits and other consumables may contain DNA, which will be present even under sterile conditions [59,141]. Once sequenced, any taxa identified in negative controls can be further scrutinized in the analysis to determine the origin of the samples [81].

Human DNA can also contaminate samples, either from the researchers or others present in the sampling area. This type of contamination has been referred to as ‘human genetic bycatch’ and raises ethical concerns. For instance, one study was able to detect some haplotypes of people present in a room from 5 h of sampling [142]. In busy public areas, it will not be feasible to obtain consent from everyone present, so it is recommended that reads aligned to the human genome are removed from datasets before publication.

### How does sequence data relate to species abundance?

A drawback of current eDNA analysis approaches is that the absolute concentration of taxa in the sample cannot be accurately inferred. In air studies, once DNA is extracted and sequenced, it is possible to calculate the concentration of DNA in the air. However, different taxa contain varying quantities of DNA, so there is no linear relationship between DNA concentration and taxon abundance as determined from sequence data.

A concern with eDNA datasets is their inherent compositional nature, meaning they reflect relative, rather than absolute, abundance. For example, a spike in one phylum’s abundance can artificially reduce the apparent abundance of others, complicating the assessment of true abundance changes. This is especially problematic in airborne longitudinal studies, where shifts in relative proportions may not reflect actual increases or decreases in the abundance of taxa, but rather changes in the overall community composition (e.g. due to the release of pollen in spring/summer). For more details on compositionality in eDNA data, see [143].

One way to overcome this compositionality could be with spike-ins of synthetic DNAs at known concentrations. These have been used to provide semi-quantitative estimates of fungal DNA concentration in air [10].

Additionally, some evidence suggests a correlation between airborne eDNA data and species abundance. For example, a study monitoring agricultural pathogens with WGS quantified pathogen abundance changes over the growing season and correlated these with environmental factors [4]. Wind tunnel experiments have also shown that increasing the number of spores released from particular taxa leads to a higher relative abundance of that taxa in sequenced samples [4]. Therefore, relative abundance can serve as a proxy for airborne spore concentration.

With enough genomic data, it should be possible to estimate population sizes of detected taxa. In aquatic environments, genomic data has been successfully used for this purpose [144]. However, the DNA concentrations in airborne eDNA samples are currently too low to achieve the genomic coverage required for such analysis. As sampler sensitivity and sequencing technologies continue to improve, it may become feasible to collect and sequence enough airborne eDNA to estimate effective population sizes of taxa. This could open new possibilities for population-level studies of airborne taxa.

### Microbial source identification

Biological particles can travel great distances depending on their size, wind speed and direction and other meteorological factors. For organisms that rely on air dispersion, such as spores and pollen, dispersal patterns have evolved over millennia. Some species remain near their origin due to favourable environmental conditions, while others travel far, possibly to reduce host density for pathogens. The factors controlling particle dispersion interact in different ways to affect particles along their trajectory before potential deposition onto an air sampler, making it exponentially difficult to ascertain the source of material using meteorological data.

Identifying the origin of airborne biological material is crucial for disease control and bioterrorism detection. For instance, if an air sampler detects foot-and-mouth disease DNA, pinpointing the farm of origin is critical for containment, a principle also applicable to plant or human diseases. Many studies infer source locations of airborne eDNA based on detected taxa, such as wheat pathogens originating from nearby wheat fields. One study used light microscopy of Hirst traps in 38 locations to track *Alternaria* spores travelling from the Pannonian plain to Poland, identifying transport events through many factors including high *Alternaria* spore concentrations at the source and the detection of atypical spores at the destination [145]. This study also applied atmospheric footprint modelling to estimate air mass trajectories before deposition onto the samplers.

Atmospheric footprint modelling relies on particle dispersion models, such as the hysplit (Hybrid-Single Particle Lagrangian Integrated Trajectory) model. hysplit uses meteorological data to determine air mass movement and to infer the transport pathways of airborne particles [146]. However, real-world application is challenging due to variables like particle size, topography, wind speed and direction, humidity and precipitation. Despite these complexities, particle modelling has been used in airborne pollen and eDNA studies to elucidate the source of airborne taxa [131,145, 147, 148]. Particle dispersion models can also be used to guide sampling location, the importance of which was discussed in Section 4.1.4.

Advances in aerobiology, including expanded sampling networks and refined dispersion modelling, are improving our ability to track microbial sources with greater accuracy. While precise pinpointing may remain difficult, integrating meteorological models with airborne eDNA monitoring will enhance source attribution and sampling strategies.

## Looking to the future

The field of airborne eDNA analysis is rapidly advancing, moving beyond slower culture-based and microscopy methods towards real-time detection techniques. Within the next few years, ongoing technological advancements will likely open new avenues for airborne eDNA studies, enhancing our understanding of microbial communities and their environmental and health implications. This section highlights key future directions for air sampling, focusing on continuous monitoring, *in situ* testing, plant pathogen monitoring networks and improvements to low-input sequencing.

Proof-of-concept studies have shown that with improved technology, air samplers could enable real-time pathogen surveillance in agricultural fields, barns or high foot-traffic zones like airports, alerting farmers or authorities to potential threats [2,23, 81]. Portable devices may also be used for personalized monitoring [37,82]. Endangered species could also be monitored using non-invasive techniques [6,20].

In healthcare settings, air samplers could help to detect and mitigate airborne disease spread in high-risk areas. For instance, deploying air monitors in wards for immunocompromised patients could enable rapid action when harmful bacteria are detected. Memon *et al*. provide an in-depth review summarizing the importance of indoor air sampling for public health and the available devices [149].

*In situ* testing in fields, hospitals and public transport is becoming more feasible with smaller hardware, lower power consumption and reduced computing requirements. A key challenge for *in situ* deployment is the automation of DNA processing and sequencing. While portable PCR and loop-mediated isothermal amplification technologies already exist, DNA extraction and library preparation are still largely confined to laboratories. Emerging devices like Oxford Nanopore Technologies’ planned TraxION and Integra Biosciences’ MIRO CANVAS represent a new generation of digital microfluidics capable of automating protocols, potentially enabling field deployment.

*In situ* sample testing will lead to faster diagnostics and rapid decision-making, eliminating delays associated with transport and laboratory processing. This is critical for responding to human health threats or plant pathogen outbreaks. Additionally, it removes the need for sample storage and transport, reducing the risk of deterioration and contamination.

Over the next decade, sequencing-based pathogen analysis could revolutionize agriculture. Fungal spore monitoring networks, already used in French vineyards [150], could be expanded to model and predict disease severity. These networks could also leverage existing air monitoring infrastructure [36], providing early warnings when emerging pathogens spread to new regions, an increasing concern due to a warming climate and increased globalization [151]. Air samplers could be deployed in warehouses storing fresh produce to monitor postharvest diseases, minimizing yield loss and preventing toxic chemical buildup in stored grain. In the future, *in situ* testing could even be integrated with precision agriculture, where air sampling data guides real-time application via direct injection mixture spray systems on tractors.

As technologies advance, with improved samplers, enhanced low-input sequencing protocols and reduced error rates, airborne eDNA analysis will become more precise, sensitive and scalable. This could enable the detection of antimicrobial, fungicide and herbicide resistant genes within pathogens [11,81, 83], strain-level taxonomic identification [24,152] and even SNP-based monitoring to measure effective population sizes, which could be especially valuable for tracking endangered species [144].

Interdisciplinary collaboration and technological innovation will be key to unlocking the full potential of airborne eDNA for real-time monitoring. Advances in sequencing accuracy, reference databases and source-tracking models will enhance reliability. Additionally, addressing challenges to *in situ* processing, viability assessment and detection sensitivity will further expand its applications in health, agriculture and environmental monitoring.

## Abbreviations

AMR: antimicrobial resistance
ASVs: amplicon sequence variants
EC: electrostatic collector
eDNA: environmental DNA
hysplit: Hybrid Single-Particle Lagrangian Integrated Trajectory
ITS: Internal Transcribed Spacer
LCA: lowest common ancestor
MAGs: metagenome-assembled genomes
NCBI: National Center for Biotechnology Information
NGS: next-generation sequencing
OTUs: operational taxonomic units
qPCR: quantitative PCR
SNP: single nucleotide polymorphism
WGS: whole-genome shotgun sequencing
